# Environmental Education in Initial Training: Effects of a Physical Activities and Sports in the Natural Environment Program for Sustainable Development

**DOI:** 10.3389/fpsyg.2022.867899

**Published:** 2022-03-09

**Authors:** M. Luisa Santos-Pastor, Pedro Jesús Ruiz-Montero, Oscar Chiva-Bartoll, Antonio Baena-Extremera, L. Fernando Martínez-Muñoz

**Affiliations:** ^1^Department of Physical Education, Sport and Human Motor Skills, Autonomous University of Madrid, Madrid, Spain; ^2^Department of Physical Education and Sport, Faculty of Education and Sport Sciences, Campus of Melilla, University of Granada, Melilla, Spain; ^3^Department of Education and Specific Didactics, University Jaume I of Castellón, Castellón de la Plana, Spain; ^4^Department of Didactics of Musical, Plastic and Body Expression, University of Granada, Granada, Spain

**Keywords:** sustainable development, environmental education, physical activity, natural environment, initial training

## Abstract

Training for sustainable development is an educational challenge of prime importance. Physical activity and sports in natural environments provide training committed to sustainability and environmental education. The objective of this study was to assess the effects of an undergraduate training program in Physical Activities and Sports in Natural Environments concerned with sustainable development. A total of 113 students from the Autonomous University of Madrid (Spain) who are studying a Bachelor’s Degree in Physical Activity and Sports Sciences and a Master’s Degree in Teacher Training for Secondary Education and High School (specializing in Physical Education) were involved. Specifically, we aimed to assess the impact of this training program on three dimensions related to Environmental Education (knowledge, behaviors, and attitudes). Its effect was also examined bearing in mind students’ sex, age and educational profile (sportive and educational). Mixed-methods were used: for the quantitative approach, data were collected with the Environmental Attitude in Physical Activities in Natural Environments validated scale; for the qualitative approach individual reflective diaries completed by students that attended the program were analyzed. The results show positive effects on the students regarding the three dimensions of Environmental Education, for both the sportive and educational training profiles. The quantitative results do not present significant differences concerning the gender variable, although the qualitative information shows that female students perceived a greater environmental sensitivity during their practices. Regarding the age variable, significant differences are found in the youngest students with an educational profile. To conclude, the study ratifies that the program carried out generated improvements in terms of knowledge, behaviors and attitudes toward the environment and sustainable development. Future research should use larger samples and consider other variables related to education for sustainability.

## Introduction

In the twenty-first century, caring for the planet is a pressing challenge requiring urgent attention. Climate change is a real threat that should be tackled from a multidisciplinary and pluri-disciplinary perspective. In this respect, education may be the perfect ally to respond to the socio-environmental problems of the planet (Lindsey and Chapman, 2017). Therefore, quality training of future generations emerges as a necessary tool to guarantee a more sustainable future (Biberhofer and Rammel, 2017; Rieckmann, 2017; Probst et al., 2019). This means that formal education must respond to an environmental commitment with sustainability (García-Rico et al., 2021), from an ecological, sociocultural, and economic perspective (Galtseva et al., 2020).

For this reason, the 2030 Agenda for Sustainable Development (United Nations, 2015) provides a blueprint to face current problems to end poverty, protect the planet, or guarantee peace and prosperity for people, among other purposes (Shabalala and Ngcwangu, 2021). The 17 Sustainable Development Goals (SDG) and their 169 targets specify the principles to achieve a more sustainable world to benefit people and the planet (UNESCO, 2017). In this way, an increased environmental awareness is fostered to guarantee responsible citizenship, as pointed out by Kroll et al. (2019).

Initial teacher training faculties must carry out programs that pursue the development of skills related to Environmental Education (EE) (Ferreira and Tilbury, 2012; Álvarez-García et al., 2019; Hasek and Torrres-González, 2021). On the other hand, Education for Sustainable Development (ESD) is perceived as an educational paradigm (Hobusch and Froehlich, 2021) capable of making possible a harmonious relationship between knowing about the natural environment and being committed to it. Van Rheenen and Melo (2021) recently proposed a paradigm shift based on critical ecopedagogy and focused on a weighty and systemic change for sports carried out in natural environments. Systemic sustainability is based on the notion of relational equity, eco-literacy and environmental justice (Van Rheenen and Melo, 2021, p. 8732). In this sense, Smith (2021) asserts that Physical Education (PE) practice in outdoor spaces and developed in natural environments should be leaded by the aspiration toward caring for the planet in a practical and disciplined way. Environmental immersion, thus, could be considered as a possibility for ecopedagogy (Valderrama-Hernández et al., 2020).

In this line, EE from an ESD perspective generates knowledge of the natural environment (to know), procedures or positive behaviors toward the natural environment (to do) and attitudes (to be), which respond to the desirable sustainable development. Knowledge related to concepts of the natural environment, by itself, does not generate behavioral changes. Nonetheless, there seems to be a cyclical relationship between knowledge about the environment and attitudes about it, that may lead to the development of values toward the natural environment (Östman et al., 2019).

In particular, Physical Activities and Sports (PAS) may emerge as a possibility to preserve and protect the environment, as long as they are developed and managed in a sustainable and authentic way in the educational context (Melo and Gomes, 2016; Orr et al., 2020). Specialized professional training focused on PAS must deal with sustainable development, to optimize teaching from an ecological lens. As Bai et al. (2021) put it, this means understanding the environment as an integrated and complex system, where the training process should be contextualized. Likewise, Baena-Morales et al. (2021a) consider that PE could help achieve the 2030 Agenda goals and targets, since it creates a very favorable context for the development of cooperation, respect, coeducation and entrepreneurship, which are aspects related to both ESD and the SDGs. However, different studies (Tuncer et al., 2009; Baena-Morales et al., 2021b; Bai et al., 2021) clearly report that physical practice that is respectful of nature does not necessarily generate environmental values by itself.

Leal Filho et al. (2019), for their part, propose a teacher training model based on the framework of the SDGs by combining sustainability skills with environmental emotions. Thus, as a pedagogical proposal to achieve the objectives of the 2030 Agenda, they bet on active teaching strategies, such as Service-Learning. This approach provides a service to the community that goes beyond pure action, since it generates students’ direct involvement. Therefore, Service-Learning might promote significant effects related to environmental attitudes.

On a more concrete level, Östman et al. (2019) seek to balance the use, the enjoyment and the preservation of the natural areas. In fact, Physical Activity and Sport in Natural Environments (PASNE) may adopt a comprehensive model of EE, since it emerges as an ideal strategy to achieve the desired effects in terms of education for sustainability (García-Rico et al., 2021). In addition, Baena-Extremera and Granero-Gallego (2014) highlight the relevance of emphasizing on the students’ way of thinking and acting to improve the environment. To do so, programs must be properly planned and organized. Likewise, students are expected to act as promoters of change. Therefore, students should understand the environmental needs and interests, and foster the reduction of environmental deterioration while building a socially responsible awareness (Biberhofer and Rammel, 2017).

Solís-Espallargas et al. (2019) use Miller’s pyramid to delimit three levels of skills acquisition in a training program. To present it, they focused on an ESD program aimed at impacting on EE. These levels are divided in: “knowing” (first level), “knowing how” (second level), focused on doing or behaving; and “showing and doing” (third level), related to the attitude regarding one-self action.

The study carried out by Puertas-Aguilar et al. (2021) shows that PAS teacher training aiming to promote sustainability-related skills must consider EE and the SDGs, besides of the scientific content of the subject as well as its didactics (pedagogy). In addition, for PASNE to achieve an authentic EE, the practitioner must be aware of the importance of knowing, living, feeling, and respecting natural environments.

Despite the need to promote ESD programs in initial training, research examining its effects on future PAS professionals is still scarce, as evinced in Grund and Brock’s (2018) report. In the Spanish context, to our knowledge, there are no studies analyzing the impact of a specific PASNE program on ESD. Therefore, there is a need to promote research enabling us to understand the impact that PASNE programs may have on ESD in EE. The results of these studies will let us determine the specific characteristics that a PAS program seeking to generate a perspective of sustainable development with a view focused on EE should have.

In order to approach this issue, the main objective of the present study is to assess the effects of a PASNE program from a comprehensive approach on EE for sustainable development in initial training of students enrolled in PAS degrees. To address it, the following specific objectives were specified:

(1) To analyze the effects of the PASNE program regarding the three dimensions of EE: Dimension 1. Knowledge of the natural environment and impact of the PASNE; Dimension 2. Behavioral actions toward the environment when carrying out PASNE; Dimension 3. Attitudes, values and rules when carrying out PASNE.
(2) To value the effects of the PASNE program depending on the educational profile of the students: sportive and education.
(3) To determine the relationship between the effects of the PASNE program regarding the three dimensions depending on students’ gender and age.

## Method

### Design

To tackle the study’s objective, a mixed research approach was used (Anguera et al., 2018) through a concurrent triangulation design (Creswell et al., 2003). Quantitative and qualitative data were collected to approach the specific objectives of the present study. Their complementary use let us examine the reality to be explored and understood it from a multifocal and global vision (Creswell and Plano-Clark, 2017). EE training programs are developed in complex educational contexts that require gathering different perspectives to perform rigorous analyses. Thus, a mixed methods approach emerges as a valid procedure to examine educational interventions (Malvaso and Delfabbro, 2020; Sawrikar, 2020). In the present research, data were collected during two school years (2019/20-2020/21), and participants were enrolled in the Degree in Physical Activity and Sport Sciences (DPASS).

The quantitative approach analyzed the effects on EE regarding three dimensions (knowledge, behaviors, and attitudes). In this sense, we used a quasi-experimental design, with pre-test and post-test measures to compare how participation in the program affected the students in terms of: (1) Knowledge about EE and the impact of PASNE; (2) Actions concerning EE in PASNE; (3) EE-related attitudes in the PASNE. To complement this information and better understand the results obtained in the quantitative part, an interpretative phenomenological analysis (Denzin and Lincoln, 2018). For this part, data were gathered from 113 reflective diaries which were completed by the students taking part in the PASNE subject.

### Participants

Sampling selection was intentional and not probabilistic, and was composed by students of the DPASS who had participated in outdoors practices (hiking, orienteering, cycling, snowshoeing, etc.). These students were in their second or fourth year within the degree. The subject they were enrolled in was PASNE, but it had two different profiles. The subject for second year students had a technical-sportive profile, while it was pedagogical-educational for those in fourth year. A total of 113 were involved in this study.

### Physical Activity and Sport in Natural Environments Program for Environmental Education

The program was developed in two different courses: (1) in the compulsory subject called “Physical-Sports Activities in the Natural Environment,” which was worth six credits (2 theory and practice hour for 16 weeks) and was carried out in the second semester of the second year. (2) In the optional subject called “PE Contents and Didactic Applications I: Physical Condition-Health and Activities in the Natural Environment,” which is worth six credits, and was carried out in the first semester of the fourth year.

The contents developed in these subjects positioned EE as a transversal axis. Thereby, it was approached not only theoretically, but also it was considered in all the practical sessions. Specifically, EE was linked to sports contents (orienteering, hiking, snowshoeing, cycling, rock climbing, etc.) and educational contents (designing healthy, sustainable and safe teaching and learning environments).

The sportive profile subject practices were set to initiate students in outdoors sports (hiking, orienteering, climbing, snowshoeing, etc.). This subject integrated the technical development of the modalities while considering its interaction with the natural environment. It was focused on knowing the different sports actions in the natural environment, adopting positive environmental behaviors, recognizing the possible impact generated by these practices, and acquiring attitudes, values, and rules of solidarity within different environments.

Moving now to the educational profile subject, its practices were focused on pinpointing the didactic aspects of the teaching-learning process of the PASNE. To do so, the curriculum of the different educational stages was taken as a reference. Moreover, the possibilities and limitations that schools developing this type of practices could encounter were also considered. In this case, the technical aspects of PAS activities were less relevant. Instead, the subject prioritized providing students with opportunities to experience the natural environment through globalized practices, which had to be adapted to the space where they were developed.

Both subjects applied a comprehensive model from a methodological point of view (globality, interdisciplinarity, and transversality) to develop the practices in different contexts (near, middle distance and far). Their main purpose was providing students with opportunities to know, do, feel and live the natural environment while practising PASNE. In other words, they aimed at making students aware of the need to care for the natural environment and respect it from a transformative and critical perspective. Two lecturers and an assistant taught these subjects. The practical sessions in nearby environments were carried out in the surroundings of the university campus. They lasted 1–2 h, and attempted to connect students with the natural environment in a responsible way. These sessions involved knowledge and awareness, and highlighted environmental values through playful actions, orienteering or sports initiation activities such as cycling or climbing. The practical lessons in middle distance environments entailed a search for new and uncertain natural spaces. They lasted 3–4 h, and students could improve sports techniques previously learned, as well as recognize the environmental values of the settings visited. Lastly, the far environments let students explore and discover the natural environment in its essence. They lasted 5–6 h, and students interacted with natural spaces of high ecological value where to put PASNE into practice. In addition, they could assess the impact generated with its practice. Specific transversal actions were carried out to favor EE when carrying out the different activities.

### Instruments and Procedures

To perform the present research, the following quantitative instruments were used:

For the quantitative part of the study, the Scale for Environmental Attitude in Physical Activities in Natural Environments (SEAPANE) was used (Santos-Pastor et al., 2019). The pre-test was administered at the beginning of the subject, specifically, during its first week. The post-test, on its part, was carried out when the subject finished. This scale aims to assess students’ attitudes toward the environment when practising physical activities in natural environments. The scale has 15 items with 5 response options (1-I do not agree at all; 5-I strongly agree). It specifies three dimensions related to EE:

(1) Knowledge of the natural environment and impact of PASNE. It collects the knowledge and reasons underlying the interest that the participants have regarding nature and its care, as well as being aware of the environmental impact caused by different human actions.
(2) Behavioral actions toward the environment when carrying out PASNE. It revolves around the ability to work or act for the benefit of the natural environment. Moreover, it considers if the students show initiative to apply their knowledge to care for and/or protect the natural environment.
(3) Attitudes, values and rules when carrying out PASNE. It considers students’ willingness to show positive attitudes toward the environment, and to respect the rules aimed at caring for and improving the natural environment.

The reliability of the SEAPANE was significant (α = between 0.75 and 0.69). Likewise, the KMO test (*r*_*jh*_ = 0.76) and Bartlett’s sphericity test (505.980, df. 120, *p* < 0.001) showed that the structure of the scale divided into three dimensions was suitable for scaling.

On the other hand, the subject’s learning blog was used as a data collection technique for the qualitative part of the study.

The PASNE student e-blog is a formative assessment instrument that was designed specifically for this study. It provides information regarding students’ learning process, and promotes self-regulation when they carry out the tasks. E-blogs favor reflective and critical learning processes, and allow students to transfer their learning to different situations in their daily lives. This evidence is particularly significant in practical situations related to PASNE (Santos-Pastor and Martínez-Muñoz, 2020). In order to use it, the particularities of the training context, the content worked and students’ previous experiences must be considered. Consequently, e-blogs include personal data, a brief life history related to PASNE (life periods, critical events, challenges, social and familial influences, values and personal ideology, vital topics and near future ideas). Students are expected to describe the practical sessions they attended to on different posts. They are expected to write down the basic data of the session (date, location, environment and title), describe their learning, as well as reflect on and critically analyse what happened. It also includes a journal of emotions linked to the natural environment, and a final section of proofs (photos, audio recordings, videos, etc.) and further knowledge (websites, news, research, etc.).

### Data Analysis

#### Quantitative Data

The Kolmogorov-Smirnov test was used to determine the normality of the data. This test suggests that the variables were not distributed normally. After extracting the mean and standard deviation, as descriptive statistics, the *p*-value was calculated using the Wilcoxon test for related samples in order to identify significant differences. Specifically, this repeated measurement test was used for pre-test and post-test differences between the educative profile group, sportive profile group, and both groups together regarding the SEAPANE dimensions. The effect size was calculated using Cohen’s d value. It can be interpreted as small (0.2 < *d* < 0.5), medium (0.5 < *d* < 0.8) or large (0.8 < *d*) (Cohen, 1992). The Spearman correlation coefficient was used to determine the relationships between the dimensions of the gender, age range and dimensions. The correlation can be interpreted as very weak (0 < *rp* < 0.2), weak (0.2 ≤ *rp* < 0.4), moderate (0.4 ≤ *rp* < 0.6), strong (0.6 ≤ *rp* < 0.8) and very strong (0.8 ≤ *rp* < 1). All statistical analyses were performed with the statistical package for Social Sciences SPSS (SPSS, v.23.0 for Windows, SPSS Inc., Armonk, NY, United States).

#### Qualitative Data

In a first phase, the emerging themes linked to the object of study (inductive process) were identified. Subsequently, they were related to the main theories in relation to the object of study (deductive process). To do so, a series of actions were carried out: exhaustive reading of all the data collected through the instruments, record-keeping of the ideas and reflections arisen while reading the data, searching for emerging themes and, finally, development of concepts and theoretical propositions. In the following phase, the data was coded and the understanding of the topic was refined. Those vague ideas, concepts and interpretations that were found in the beginning of the process became concrete and specific. We discarded some of them, while further developed others. To this end, the following actions were carried out: development of the coding categories, coding of all the data collected, and separation and grouping of the data belonging to each coding category in order to refine the data analysis. Lastly, the data were relativized, since we adjusted and considered the context in which they were collected.

The selection of quotes was carried out using the N-Vivo (v.12) software. Next, a code was assigned to the learning e-blogs according to the student’s profile (ALG2: second year of the DPASS undergraduate student; ALG4: fourth year of the DPASS undergraduate student).

Regarding the methodological rigor of this study, a number of criteria established by Guba and Lincoln (1989) were followed. Particularly, we took into account: credibility (i.e., triangulation of techniques and negotiation of reports), transferability (i.e., exhaustive collection of data and description of the context), dependency (i.e., description of processes and external evaluator) and confirmability (i.e., transcription of information, reflection on findings and identification of limitations). Similarly, we considered the ethical criteria for qualitative research (informed consent and confidentiality) (Flick, 2015).

## Results

### Quantitative Findings Related to the Effects of the Physical Activity and Sport in Natural Environments Program From an Environmental Education Approach

The gender, age range, attendance rate of lessons and previous experience of volunteering or SL of the participants are shown in Table 1. The sample was mostly male with the 42.9% of the participants in the educative profile group, 72.6% in the sportive profile group and 69.5% on both groups together. Similarly, most of the participants were ranged between 18 and 20 years in the educative profile group (42.9%), the sportive profile group (58.4%), as well as in both groups together (55.3%). More than 90% of participants attended regularly to the lessons (75, 82.3, and 80.9%, respectively), and the percentage of participants with previous experience on volunteering or Service-Learning were higher in the educative profile group (64.3%), while the sportive profile group and the complete sample showed higher percentage of participants with no previous experience (77.9 and 69.5%, respectively).

**TABLE 1:** Sociodemographic characteristics of the participants.

	Educative profile group (*n* = 28)	Sportive profile group (*n* = 113)	Both groups together (*n* = 141)
**Gender**			
Female	12 (57.1%)	31 (27.4%)	43 (30.5%)
Male	16 (42.9%)	82 (72.6%)	98 (69.5%)
**Age range**			
18–20	12 (42.9%)	66 (58.4%)	78 (55.3%)
21–22	11 (39.3%)	31 (27.4%)	42 (29.8%)
23–24	2 (7.1%)	10 (8.8%)	12 (8.5%)
>25	3 (10.7%)	6 (5.3%)	9 (6.4%)
**Attendance rate**			
Has not attended class	–	–	
Less than 25%	–	–	
Between 25 and 50%	–	–	
Between 50 and 75%	2 (7.2%)	11 (9.7%)	13 (9.2%)
Between 75 and 90%	5 (17.8%)	9 (8%)	14 (9.9%)
More than 90%	21 (75%)	93 (82.3%)	114 (80.9%)
**Previous experience of volunteering or SL**			
With previous experience	18 (64.3%)	25 (22.1%)	43 (30.5%)
No previous experience	10 (35.7%)	88 (77.9%)	98 (69.5%)

*SL, Service-Learning.*

The results obtained from the analysis of the qualitative data extracted from the e-blogs were structured regarding the three dimensions, in coherence with the learning competency of the PASNE for ESD for EE: Dimension 1. Knowledge of the natural environment and impact of the PASNE; Dimension 2. Behaviors on the environment when carrying out PASNE; Dimension 3. Attitudes, values and rules when carrying out PASNE. Likewise, a second analysis criterion was established depending on students’ training profile: educational and sportive.

*Dimension 1. Knowledge of the natural environment and the impact of PASNE.*

#### Educational Profile

It is a real shame to walk and find plastic, cans, paper tissues and many other things on the ground. This is one of the biggest causes of the deterioration of the natural environment, and it generates problems for the living beings living there (girl) (ALG4-3).

I understand that activities in the natural environment are all those that respect nature, avoiding damaging plants or animals at all times, always respecting the land without forcing it to change, and, above all, avoiding pollution of all kinds, whether acoustic, residual, geographical, etc. (ALG4-21).

PASNE is sustainable from an environmental point of view when we are respectful of nature. This is fostered when we do not throw waste to the ground, we collect the rubbish we see, even if it is not ours, and also when we avoid noise pollution (ALG4-6).

#### Sportive Profile

I think that since I was little, I have been brought up to be concerned about respecting nature. Small habits such as not throwing rubbish or taking care of the fauna and flora of the environment (without endangering or damaging it) contribute to keeping a clean environment. Indeed, it is the responsibility of everyone to keep it as it is. I am very aware of the fact that all that nature and beauty does not stand alone, and if we want to continue enjoying it, we have to take care of it (ALG2-9).

Table 2 shows significant differences between the three EAPANE dimensions of the Educational profile’s group (D1, *p* = 0.015; D3, *p* = 0.002), Sport profile’s group (D1, *p* = 0.001; D2, *p* = 0.009; D3, *p* = 0.001) and Both groups together (D1, *p* = 0.001; D2, *p* = 0.006; D3, *p* = 0.001) except the D2 in the Educational profile’s group.

**TABLE 2:** Analysis of Scale for Environmental Attitude in Physical Activities in Natural Environments (SEAPANE) in educative profile, sport profile, and both groups together.

	Educative profile group (*n* = 28)				Sportive profile group (*n* = 113)				Both groups together (*n* = 141)			
SEAPANE dimensions	Pre-test	Post-test	*p*	*d*	Pre-test	Post-test	*p*	*d*	Pre-test	Post-test	*p*	*d*
D1: Knowledge of the NE	3.86 (0.42)	4.23 (0.49)	0.015*	–0.81	3.86 (0.54)	4.21 (0.45)	0.001***	–0.71	3.86 (0.53)	4.22 (0.45)	0.001***	–0.73
D2: Association with the action	3.85 (0.39)	4.01 (0.51)	0.280	–0.35	3.91 (0.51)	4.06 (0.48)	0.009**	–0.31	3.91 (0.48)	4.05 (0.49)	0.006**	–0.28
D3: Attitudes, values and rules stemming from NE	4.07 (0.53)	4.53 (0.52)	0.002**	–0.87	4.27 (0.52)	4.66 (0.35)	0.001***	–0.87	4.23 (0.52)	4.64 (0.38)	0.001***	–0.90

*p < 0.05, **p < 0.01, p < 0.001***.

*d, Cohen’s d; D1, Dimension 1; D2, Dimension 2; D3, Dimension 3; NE, Natural Environment.*

Regarding the relationship of the analyzed variables, it was significant in two of 18 cases. Those two cases had a level of significance of *p* < 0.01 regarding the relationship between the Dimension 2 and Dimension 3 of the educative profile group with the age range (Table 3).

**TABLE 3:** Spearman correlation between the dimensions of the Scale for Environmental Attitude in Physical Activities in Natural Environments (SEAPANE), gender and age range of the educative and sportive profile groups, and both groups together (post-test).

SEAPANE Dimensions/Gender/Age range	Educative profile group			Sportive profile group			Both groups together		
	D1	D2	D3	D1	D2		D3	D1	D2
Gender	–0.009	0.302	–0.115	0.161	0.105	0.104	0.120	0.134	0.035
Age range	0.230	0.589**	0.554**	–0.102	–0.026	0.002	–0.023	–0.053	0.116

**p < 0.01.

*D1, Dimension 1; D2, Dimension 2; D3, Dimension 3; NE, Natural Environment.*

My family has always played a fundamental role when it comes to my relationship toward natural environment. They have always taken me on excursions or to walk in the countryside since I was little. Also, we went cycling all together to the country near my town (ALG2-1).

*Dimension 2. Behaviors on the environment when carrying out PASNE.*

#### Educational Profile

Whenever one engages in PASNE, nature must be protected, and it must not be damaged nor dirtied under any circumstances (ALG2-9).

In addition, a specific group was in charge of the environmental mission, which consisted of collecting the rubbish they found and putting it in a bag along the way of our walk (ALG4-3).

#### Sportive Profile

For physical activity and sports in the natural environment to be sustainable, I consider that the characteristics of that natural environment should not be harmed, since this would endanger it for the future (ALG4-4).

As long as you respect nature, the environment and your own safety, carrying out PASNE is always recommended (ALG4-12).

*Dimension 3. Attitudes, values and rules when carrying out PASNE*

#### Educational Profile

Bearing in mind my personal experience, I must say that the environment must be protected, since it is something that brings a lot of peace to the crazy world we live in. Currently, many forests are burned, and many disasters come as a result of people’s behaviors, nobody does anything. Is it so difficult for each one of us to do a little bit to avoid this to happen? (ALG4-1).

The values that I have been taught regarding natural environment are related to the fact that one has to take care of it, keep it clean and never damage it (girl) (ALG4-6).

It is true that one of the feelings that appeared to me on several occasions is that of shame and disbelief when finding so much rubbish lying on the field. This made me feel sad when I think what we are capable of doing (girl) (ALG4-8).

#### Sportive Profile

I believe that these activities are not only useful to carry out physical activity, discover new sports and interesting activities, but also to raise awareness about caring for the planet. When we visit the natural environment, as its name indicates, it is nature, and human beings are those who may protect nature or destroy it. So, thanks to these activities, we can raise awareness and make the world a little better by caring for it together (ALG2-10).

I think it is essential that we preserve natural settings and take care of them, since we all love to enjoy nature. It provides us with many landscapes and places where we can go and carry out this type of activities. Besides, we can enjoy them in a pleasant way or relax by ourselves, with family or friends (ALG2-28).

## Discussion

The effects of a PASNE program in PAS degrees on EE for sustainable development, that carried out using a comprehensive approach, were assessed. The discussion has been structured considering the effects of the PASNE program on three dimensions of EE: (1) Knowledge of the natural environment and impact of PASNE; (2) Behaviors on the environment when practising PASNE; and (3) Attitudes, values and rules when practising PASNE. In addition, we assessed the effects of the PASNE program considering the student’s training profile (sportive and educational), and we determined the relationship between the effects of the PASNE program regarding the three dimensions, depending on the gender and age of the students.

According to the results of the study, the PASNE training program came with positive effects. This improvement appears for both, students with sportive and educational training profiles. Likewise, a positive influence is observed in the three dimensions of EE. These outcomes are supported by the statistical results obtained, through which we compared the post-test scores of both groups.

These results are similar to those reported in the study by Tikka et al. (2000), in which students who took subjects related to EE at university had greater environmental knowledge and more respectful attitudes or behaviors toward the environment. Furthermore, this result may be explained by the fact that people who carry out activities in natural environments shows greater environmental awareness (Tuncer et al., 2009; Smith, 2021). Therefore, it seems that students who receive specific EE training are more environmentally literate (they have greater environmental knowledge) than students without this training (Álvarez-García et al., 2019).

Similarly, the program produced significant differences in the three dimensions considering the students’ training profile. The dimension concerning natural environment knowledge and impact of practice, as well as the dimension focused on attitudes, values and rules regarding the environment are significant in the educational profile, in line with Östman et al. (2019). These authors affirm that there is a relationship between knowledge and attitude toward the environment in the natural context. However, in this group, no significant change in behavior in the natural environment was observed when carrying out PASNE. In any case, if we focus on the sportive profile, there was a significant improvement in the three dimensions. The effect on the modification of environmental behavior when during PAS practice in natural environments is particularly salient, even more bearing in mind that this effect is not relevant for the educational profile students. This may be explained because in the sportive profile subject, the amount of hours of PASNE activities in direct contact with nature is higher. Therefore, greater first-hand experience in the environment may have been generated. In fact, the study by Baena-Morales et al. (2021b) also reinforces the correlation between attitudes, the number of activities related to nature and knowledge about the environment. According to these authors, the longer students stay in the natural environment, the greater acquired knowledge and sensitivity to care for the natural environment developed. Likewise, this reasoning can be related to the study by Welch et al. (2021), who detected a difference in students who had taken a subject related to EE as part of the study plan at one of the universities. These students showed greater environmental knowledge, more responsible attitudes toward the environment and a more respectful behavior. The effect size showed in the sportive profile group corroborates the impact of the three SEAPANE dimensions on these students.

On the other hand, if we analyse these dimensions considering the gender variable, the results did not show significant differences in neither the educational profile nor the sports profile, despite the fact that the number of male students was higher than that of female in both profiles (69.5%). These quantitative results disagree with those reported in the study by Tikka et al. (2000), who found significant variations among students depending on their gender. This information may therefore be confusing and should be reviewed in future research. In their case, women tended to show more responsible behavior toward the environment than men. However, the information gathered from the learning e-blogs of the subject, showed that female students value their learning during the PASNE practice to a greater extent due to the improvement regarding their environmental sensitivity:

[I admit that when hiking, climbing, doing orienteering or canoeing, in addition to learning how to do them, I like to feel the natural environment in a special way, smell it, breathe it or stare at it (ALG4-10)].

On the contrary, male students show lesser concern about responsible behavior with the environment and give greater importance to the technical learning of the specific sports activity:

[I like to focus on the technical part that I have to learn and how to adapt to the natural environment (ALG2-14)].

In addition, the more technical the activity is, the less they consider the environmental consciousness:

[If I go uphill when cycling, I am more concerned about the effort and being able to move forward, than watching what I may find or not in the surrounding natural environment (ALG2-15)].

This finding is in accordance with previous studies; thus, confirming that women show greater sensitivity and environmental awareness (Larson et al., 2010). In this sense, it seems that PASNE practice from an ecopedagogical perspective is less sportive and may be effective in terms of attitudinal learning linked to environmental awareness and environmental preservation, as suggested by Smith (2021). From this viewpoint, PASNE practice itself takes a backseat, while the main focus is shifted toward ESD and EE. Consequently, it would be advisable to offer learning environments for PASNE practice, promoting balanced relationships between people and the environment from a rational critical pedagogy (Tinning, 2020).

Regarding the age variable, there are significant differences concerning students’ behavior toward the environment and their attitudes, values and rules during sports practice for the younger students who take the subject of the educational profile.

[I really value what I learned with my family. even at school, since they took us to the park to do orienteering and made us leave everything clean. but I see a difference with my older brothers who don’t pay so much attention to taking care of nature. they prefer playing sports, while they don’t care so much about the rest (ALG4-3)].

However, this result might be attributed to the fact that the percentage of young students is higher in the range of 18–20 years (55.3%). Likewise, a greater focus and awareness is perceived in their behavior when practising PASNE than in the attitude they adopt when carrying it out. This outcome coincides with the results of the study by Grund and Brock (2018). Such a difference may be explained because of the fact that the students consider that they practice PASNE in their leisure time, which allows them to apply what they learn at university and reinforce their experiences:

[I try to repeat the treks I do in the subject with friends or family (ALG4-5)].

[Since I did the PAS Vocational Training course, I enjoy going out to natural scenarios and I do it whenever I can… there are people who like to go to the gym or play soccer, but I like to explore nature and enjoy it (ALG2-15)].

On the contrary, older students usually combine their studies with working activities. Thus, they have less time to do sports in the natural environment in their free time:

[I can do these activities at the university only, because I work on weekends and I don’t have time to go to the natural environment (ALG2-3)].

[When I started the degree, I had more time and went out all day. Now I work; so, whenever I can do something, I do the activity and that is it, I don’t think about the environment or anything (ALG4-16)].

This finding again evinces the relationship between attitudes toward the environment and the levels of PASNE practice (Tuncer et al., 2009). However, it disagrees with other studies that show that students’ autonomy makes them more active. Therefore, they are expected to engage in a higher number of PASNE, which improves their positive attitudes of caring for the environment (Orr et al., 2020).

However, regarding the dimension focused on knowledge about the environment, no significant differences were obtained depending on the age of the students in either of the two training profiles. These results go in line with ta previous study carried out with high school students with similar profile (Hausbeck et al., 1992), in which no correlation was found between attitude and knowledge about the natural environment. The authors considered this was a consequence of the fact that the attitude toward the environment depends on feelings and values, as well as factual knowledge. However, other studies challenge this theory, as they show that greater knowledge produces greater interest, so this could generate a change in attitude toward the environment (Welch et al., 2021). It has not been possible to confirm whether these differences based on age, sex or educational profile can be attributed to other factors such as the influence of the educational programs that students had carried out in the previous educational stages, the experiences promoted by the family or their belonging to sports clubs. What does seem to be clear is that a PASNE training program must consider the attitude toward the environment as a priority to promote ESD.

## Conclusion

Bearing in mind the results of this study, we can conclude that a PASNE program, using a comprehensive approach and focused on EE, produced significant improvements in knowledge, behaviors and attitudes toward the environment (Baena-Morales et al., 2021b). Likewise, in line with different authors (Mezirow and Taylor, 2009; Sterling, 2011), an effective training program that aims to influence sustainable learning should contemplate three perspicuous levels: (1) Knowledge and/or awareness of the environment; (2) Beliefs, values, assumptions and ways of acting toward the environment; and (3) Understanding toward the environment fostering the transformation of behaviors and attitudes. This last aspect may be considered as an important basis for didactics of PASNE focused on the EE field and aiming to develop sustainability competence.

In addition, this study shows that PASNE practice has a relevant role in sustainability and in the acquisition of quality EE. Therefore, we highlight its relevance and demand its inclusion in the curriculum at different educational stages. In addition, the teaching staff responsible for implementing these programs should be encouraged to take part in professional training courses and innovation programs that help achieve environmental literacy for PAS and thus, contribute to ESD promotion with reference to the SDGs (Baena-Morales et al., 2021c; Shabalala and Ngcwangu, 2021).

## Limitations

Regarding the limitations of this research, it is necessary to take into account that the study was carried out in a Spanish University and in its specific training programs. As a result, the sample was not representative and, therefore, the results cannot be generalized. Furthermore, sample selection was not random. Likewise, despite the fact that a mixed research design was used, it has not been possible to delve into some features of the training program that may have caused different effects in students’ sustainability training. It would be desirable to use other instruments to collect more complete information, whether quantitative (assessment scales or checklist) or qualitative (observation scale and interviews with the main actors). In addition, future studies could extend the sample to other Universities offering the DPASS to assess the effectiveness of the PASNE programs from an EE perspective for sustainable training. Similarly, the discourse of the participant teachers and students could be explored to collect different views on initial training for sustainable education.

## Data Availability Statement

The original contributions presented in the study are included in the article/supplementary material, further inquiries can be directed to the corresponding author.

## Ethics Statement

The studies involving human participants were reviewed and approved by the Comité de Ética de la Universidad Autónoma de Madrid. The patients/participants provided their written informed consent to participate in this study.

## Author Contributions

MS-P, LM-M, OC-B, PR-M, and AB-E contributed to the conception and design of the study and wrote sections of the manuscript. MS-P organized the database. PR-M performed the statistical analysis. MS-P wrote the first draft of the manuscript. All authors contributed to the manuscript revision, read, and approved the submitted version.

## Conflict of Interest

The authors declare that the research was conducted in the absence of any commercial or financial relationships that could be construed as a potential conflict of interest.

## Publisher’s Note

All claims expressed in this article are solely those of the authors and do not necessarily represent those of their affiliated organizations, or those of the publisher, the editors and the reviewers. Any product that may be evaluated in this article, or claim that may be made by its manufacturer, is not guaranteed or endorsed by the publisher.
